# Comprehensive Geriatric Assessment for Older Women with Early-Stage (Non-Metastatic) Breast Cancer—An Updated Systematic Review of the Literature

**DOI:** 10.3390/curroncol30090602

**Published:** 2023-09-07

**Authors:** Chantae Reid-Agboola, Anita Klukowska, Francesca L. Malcolm, Cora Harrison, Ruth M. Parks, Kwok-Leung Cheung

**Affiliations:** 1 Nottingham Breast Cancer Research Centre, University of Nottingham, Nottingham NG7 2UH, UK; mzycr3@exmail.nottingham.ac.uk (C.R.-A.); msaak21@exmail.nottingham.ac.uk (A.K.); francesca.malcolm@nhs.net (F.L.M.); cora.harrison1@nhs.net (C.H.); ruth.parks@nottingham.ac.uk (R.M.P.); 2 School of Medicine, University of Nottingham, Royal Derby Hospital Centre, Uttoxeter Road, Derby DE22 3DT, UK; 3 Royal Free Hospital, Royal Free London NHS Foundation Trust, London NW3 2QG, UK

**Keywords:** comprehensive geriatric assessment, breast cancer, primary, operable

## Abstract

Background: A previous systematic review by our team (2012) undertook comprehensive geriatric assessment (CGA) in breast cancer and concluded there was not sufficient evidence to instate CGA as mandatory practice. SIOG/EUSOMA guidelines published in 2021 advocate the use of CGA in breast cancer patients. The aim is to perform an updated systematic review of the literature. Methods: A systematic review of studies published between 2012 and 2022 that assessed the use of CGA in breast cancer was performed on Cochrane, PubMed and Embase. Results: A total of 18 articles including 4734 patients with breast cancer were identified. The studies covered four themes for use of CGA in breast cancer: (1) to determine factors influencing survival (2) as an adjunct to treatment decision-making (3) to measure quality of life, and (4) to determine which tools should be included. There was evidence to support the use of CGA in themes 1–3; however, it is uncertain which assessment tools are best to use (theme 4). Conclusions: CGA can be used to determine factors affecting survival and quality of life in breast cancer patients and can therefore be used to aid treatment decision-making. Further work is required to determine gold standard CGA.

## 1. Introduction

Breast cancer is the most common cancer afflicting women worldwide, with approximately 24% of new cases diagnosed each year in women aged over 70 years [1]. Despite this, most research is focused on younger women [2]. Older patients tend to be frailer and have greater comorbidities, which can affect treatment decisions [2].

The recommended treatment for all patients with breast cancer is surgery; however, in more frail patients with oestrogen receptor-positive breast cancer, primary endocrine therapy (PET) may be offered as an alternative [3]. Despite the latest guidance from the International Society of Geriatric Oncology (SIOG) advising that PET should only be given to women with a life expectancy of <3 years [4], the latest National Audit of Breast Cancer in Older Patients (NABCOP) in the UK reported in 2020 that around 25% of all women with breast cancer aged ≥70 years had non-operative treatment [5]. NABCOP suggests that a fitness assessment should be conducted for older women to help determine their suitability for treatment [5].

The SIOG guidelines state that clinicians should be routinely assessing for frailty in all breast cancer patients to allow for a starting point for further discussion and assessment, for example, by conducting a comprehensive geriatric assessment (CGA) [6,7]. CGA typically includes assessment of the following domains: functionality, nutrition, cognition, psychological state, social support, comorbidities, medications, and geriatric syndromes [7,8]. However, there is currently no consensus on what constitutes the ‘gold standard’ CGA. Ultimately, a universally accepted standardised CGA model to implement in breast cancer patients is yet to be determined [7,8].

A previous systematic review conducted by our team in 2012 analysed the use of CGA in older women with early breast cancer [8]. This review identified nine studies that reported on the utility of CGA in regard to functional status assessment, prediction of chemotherapy toxicity and recognition of the impact of pre-existing comorbidities on treatment. There was a paucity of high-level data [8].

This present review has been carried out to reassess the evidence for use of CGA in older patients with early breast cancer. It is anticipated that over the past decade that developments in this field have been made, highlighted by SIOG guidelines published in 2021 [4]. Our aim is therefore to evaluate literature published between 2011 and 2022 concerning the use of CGA in older patients with early breast cancer.

## 2. Materials and Methods

### 2.1. PRISMA Statement

This systematic review was conducted in accordance to the guidelines outlined in the Preferred Reporting Items for Systematic Reviews and Meta-analyses (PRISMA) statement [9].

### 2.2. Search Strategy

**Figure 1:**
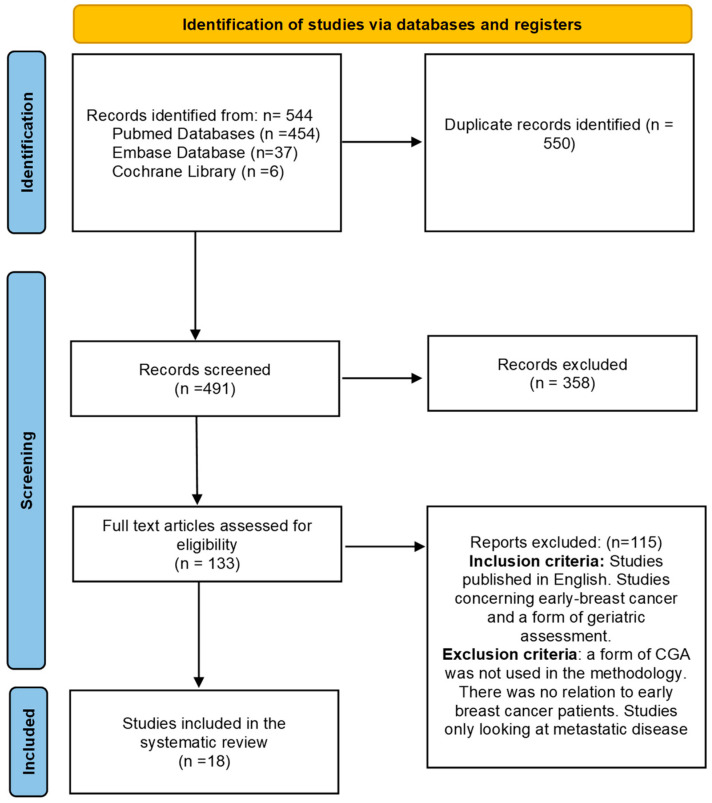
PRISMA flow diagram of systematic review literature search.

The search was conducted using the databases PubMed, Embase and Cochrane Library (Figure 1). Literature published between September 2011 and June 2022 was searched according to the methodology described by Parks [10] and utilised the search terms ‘comprehensive geriatric assessment’, ‘breast cancer’, ‘primary’ and ‘operable’.

### 2.3. Inclusion and Exclusion Criteria

Studies published in English that addressed the use of CGA in early breast cancer patients were included. Studies were excluded if a form of CGA was not used in the methodology or there was no relation to early breast cancer patients.

### 2.4. Study Selection

The abstracts of studies identified within the search were screened by two independent researchers (CRA, CH). Relevant full-text articles were reviewed. Any discrepancies were resolved by discussion by a third reviewer (RP).

### 2.5. Data Extraction

The following data were extracted from the studies meeting the inclusion criteria: ‘country of study origin, ‘date’, ‘lead author’, ‘aims of analysis’, ‘study type’, ‘level of evidence’, ‘number of participants’, ‘age of participants’, ‘cancer type’, ‘stage of cancer’, and ‘tools used’. No additional statistical analysis was performed.

### 2.6. Critical Appraisal

All studies were assessed for their level of evidence using the system proposed by the US Agency for Healthcare Research and Quality, which has been used in the recent 2021 SIOG recommendation on breast cancer management in older patients [11]. Studies are awarded a level of evidence score from I to IV (Table A1 in Appendix A). All of the studies included in the review were above level IV, which is an expert opinion. There were 17 studies included in this review that were awarded level III, due to the descriptive nature of the studies. There was one study awarded level II, which was a cross-sectional study. There were no level I studies, which is awarded to randomised clinical trials, included in this review after our inclusion and exclusion criteria were applied [11].

## 3. Results

### 3.1. General Characteristics

The search for studies was completed on 1 July 2022. The initial search retrieved 554 articles (Figure 1) and 18 met the inclusion criteria. The characteristics of the included studies are shown in Table A1. There were 13 studies that conducted CGA pre-treatment [12,13,14,15,16,17,18] and six studies that conducted CGA post-treatment [19,20,21,22,23]. A total of 17 studies included patients >65 years of age or older. One study selected patients based on frailty rather than age, and hence the youngest patient included was 43 years old [12].

Varied CGA methodology was reported. Table A2 provides a summary of the CGA domains examined across the studies and the tools utilised to assess these domains. Overall, eight CGA domains were represented and a total of 24 assessment tools were reported. The most frequently reported domain was functionality, and the most utilised tool was the assessment of ‘instrumental activities of daily living’ (I-ADL).

### 3.2. Level of Evidence

In sum, 17 studies were graded level III [12,14]. One paper was awarded level IIa due to it being a quasi-experimental clinical study [13].

### 3.3. Findings

The studies were categorised into four main themes based on their aim: (1) to determine factors influencing survival or mortality, which comprised five papers [12,13,14,15,19], (2) as an adjunct to treatment decision-making, which comprised six papers [16,17,20,21,22,23] (3) to measure quality of life and functional status, which comprised four papers [18,24,25,26], and (4) to determine which tools should be used in CGA, which comprised three papers [27,28,29]. Further discussion about the findings of studies and future directions are highlighted in Table A3.

### 3.4. To Determine Factors Influencing Survival or Mortality

Two studies utilised CGA alone to predict survival or mortality in breast cancer patients. Stotter, A. et al. [12] recommended the use of CGA pre-treatment to help indicate survival at 3 years. Clough, K.M. et al. [13] found that those with deficits in three or more domains on CGA had a twice the breast-cancer-specific-mortality rate at 5 and 10 years of women who had fewer deficits.

Guerad, E.J. et al. [14] analysed whether CGAs in cancer patients appropriately identified falls risk and found that falls were found to be an indicator of survival rate amongst cancer patients.

Two studies demonstrated the utility of combining CGA with other parameters to determine mortality. Liuu, E. et al. [15] concluded that multidimensional prognostic index (MPI) alongside CGA was able to predict mortality at 12 months for breast cancer (and other cancer types). Speigl, L. et al. [19] used a CGA combined with immune markers to determine mortality in breast cancer patients. Patients deemed ‘fitter’ on CGA had higher levels of infiltrating CD3+ cells and a lower 5-year mortality.

### 3.5. As an Adjunct to Treatment Decision-Making

CGA was used to determine risks and benefits of treatment or to influence treatment decisions in six studies. Okonji, D.O. et al. [20] found that breast cancer patients who were considered ‘fit’ (having ≤1 than or equal to CGA domain deficit) were more frequently offered surgery. Three studies utilised CGA in the context of determining tolerance of chemotherapy. Bailur, J.K. et al. [21] found that breast cancer patients identified as frail on CGA were at greater risk of adverse reactions to chemotherapy. Similarly, Freyer, G. et al. [22] demonstrated that CGA could be used to predict chemotherapy toxicities. Blanc, M. et al. [16] analysed use of CGA on cancer treatment recommendations versus recommendations from standard ‘multi-disciplinary team’ (MDT) or ‘tumour board’ discussions. It was found that patients were less likely to be offered chemotherapy following CGA due to identified risk of adverse outcomes. Falandry, C. et al. [17] surveyed clinicians treating older patients with breast cancer and found that 61% based treatment recommendations on performance status measured by CGA. Denkinger, M.D. et al. [23] compared the use of CGA versus other cancer screening assessments to determine the adverse reactions from radiotherapy in breast cancer patients and concluded that CGA was the best predictor of fatigue following radiotherapy.

### 3.6. Measuring Quality of Life and Functionality

Boulahassass, R. et al. [18] used QLQ-C30, QLQ-BR23 and CGA to assess QoL in breast cancer patients receiving radiotherapy post breast surgery. There were no significant differences observed for functional items (physical, emotional, cognitive, and social) at 1, 3 or 6 months. Quinten, C. et al. [24] identified that there was a strong correlation with a poor CGA baseline score and the self-reported QoL. This study also identified that QoL deteriorated over the course of breast cancer treatment. Parks, R.M. et al. [25] found no correlation between a patient’s QOL score and whether the patient was offered surgical treatment. The study confirmed the feasibility of being able to conduct a CGA in a research setting. Owusu, C et al., 2013 [26] used CGA to determine functional disability, defined as any dependency with activities of daily living (ADLs) in breast cancer patients. Functional disability was prevalent within the cohort study and disproportionately higher in African American patients.

### 3.7. To Determine Which Tools Should Be Used in CGA

Biganzoli, L. et al. [27] concluded that cardiovascular health score (CHS) was more accurate as a screening tool than the Vulnerable Elderly Survey-13 (VES-13), for comparison to CGA outcomes. Owusu, C et al., 2018 [28] reported a range of tools used in CGA (Table A1) that were successful in predicting physical performance of patients. Munir, A. et al. [29] concluded that self-administered CGA might influence treatment decisions by highlighting specific morbidity that could influence the use of chemotherapy and radiotherapy.

## 4. Discussion

Our results have demonstrated that CGA can be successfully used to predicting outcomes and in the assessment of QoL in breast cancer patients. We have also provided an updated insight into an array of assessment tools that are available. However, it is still unclear which tools are optimal to be used for a CGA.

### 4.1. Level of Evidence

In sum, 17 of the studies were awarded level III and one study was level II [11]. There is a lack of level I evidence related to CGA use in studies of patients with early breast cancer, and hence a gap is still evident within the current literature [4].

### 4.2. Factors Influencing Survival or Mortality

There were five studies that were focused on the use of CGA for predicting the survival or mortality rate of patients with breast cancer, either as a standalone metric or in combination with other measures [12,13,14,15,19]. Liuu, E et al. presented a novel use of CGA in combination with MPI to predict mortality at one year following cancer diagnosis. Notably, this study included multiple cancer types, 12% of which had breast cancer and hence may not be of specific relevance. Speigl, L. et al. [19] successfully showed how CGA can be used in conjunction with intra-tumoural CD3+ and CD15+ leucocytes as potential biomarkers to help predict the mortality of breast cancer patients post-treatment. The ability to estimate mortality following an intervention is extremely important in the setting of breast cancer at diagnosis, where a range of treatment options exist [30]. Although breast cancer surgery is deemed less morbid than surgery for other types of cancers, other studies have shown that functional status does decline after breast cancer surgery and severity of decline is associated with extent of surgery [31].

In a study assessing CGA in patients with renal carcinoma, Pierantoni, F. et al. [32] further highlights how CGA can be used to predict survival. This study used CGA to identify the fitness level of patients to determine what treatment would optimise their survival rate. Patients were categorised into three categories from their CGA (fit, vulnerable, or frail), which indicated their chances of survival and adverse effects from treatment. These findings are akin to those reported by Stotter A. et al. [12], where CGA was also used to aid treatment decision-making based on predicted survival rate of patients.

In summary, the use of a CGA to help determine survival rate or mortality in breast cancer patients can be achieved through a variety of methods.

### 4.3. As an Adjunct to Treatment Decision-Making

There were six studies investigating CGA as a tool to optimise treatment choice for patients with breast cancer [16,17,20,21,22,23]. Okonji, D.O. et al. [20] used CGA to determine suitability of treatment options [23]. In Falandy, C. et al. [17], 39% of treatment plans that were recommended by oncologists were changed following a CGA. Similarly, Blanc, M. et al. [16] highlighted the differences in treatment recommendation following CGA versus usual ‘MDT’ recommendation. This demonstrates the utility of CGA in the assessment of a patient’s suitability to proceed with proposed treatment. The importance of involving patients in the decision-making process was indicated in Parks, R.M. et al. [25]. This study also showed a potential trend: with an increasing age, patients were less likely to opt out of receiving aggressive treatment.

The studies reviewed have highlighted how CGA can also be used to help predict the optimal treatment decision for patients.

This conclusion was also observed in additional studies: Bai, J.F. et al. [33] and Sourdet, S [34]. In Bai, J.F et al., the study focuses on predicting the best treatment options for patients who have large B-cell lymphoma after having a CGA [33]. The reported use of CGA to influence appropriateness of chemotherapy as a treatment option [33] for patients is similarly seen in Okonji, D.O. et al. [20]. Sourdet, S et al. concluded that patients who have a high score in the CGA domains (physical, psychological and nutrition) are associated with having a change in their treatment plan due to the predicted mortality outcome post-treatment, thus providing further evidence of how beneficial a CGA can be for treatment decisions for patients [34]. Studies have also highlighted the effect of patient’s social support systems can also play into effect on treatment decision-making. Those patients who reported having more social support were also associated with better psychological adjustments to their cancer diagnosis [35,36]. Further research needs to be conducted, such as a prospective study that investigates the hesitancy of patients choosing more aggressive treatment, despite their CGA concluding that they would be able to tolerate it, as seen in the studies by Boulahssass, R. et al. [18] and Lawhon, V.M. et al. [37].

CGA can be used as an adjunct to treatment decision-making in breast cancer patients and can help tailor a patient’s treatment regime.

### 4.4. To Measure Quality of Life and Functional State

There is still no gold standard QoL tool that is used in a full CGA, yet QoL is an important factor in determining the treatment options for patients with breast cancer. Breast surgery has a huge impact on a patient’s psychological well-being as well as physical impairments. It is also important to regard how alternative treatment, such as chemotherapy, would have an impact on a patient’s functional status; the patient may want to consider preservation of QoL instead of prolonging life.

Perry, S. et al. [38] reviewed the literature based on QoL assessment in breast cancer patients and concluded that QoL assessment was beneficial to aid delivery of holistic, patient-centred care. Perry, S et al. also noted the need for standardisation across QoL assessment. This is in line with the results from the studies identified in this review: there was no gold standard QoL assessment that is best used alongside a full CGA in older patients with early breast cancer [38].

### 4.5. To Determine Which Tools Should Be Used in CGA

None of the studies definitively concluded which tools should contribute towards CGA; however, from reviewing the evidence provided, all studies assessed at least the functional status, physical status, and psychological status of the patients. This provides sufficient evidence that a CGA should at a minimum include tools that assess the domains functional status, physical status and psychological status. Over 20 were tools used, with some studies using multiple tools to assess the same domain. It was not clear as to why this was the case, and hence we were unable to determine a gold standard method in which a CGA can be performed within the context of breast cancer patients.

A screening tool is most often used before a full CGA is conducted, as it allows clinicians to identify the vulnerable patients and then proceed with the rest of the CGA. There were 13 studies that used a CGA screening tool as per the European CGA model. Based on methodology, it was not clear as to why some studies chose to focus on utilising some tools over others, as this was not mentioned in their methodologies. In an additional study, Torres-Hernadez, C. et al. [39], they recommended as a minimum that ADL/iADL should be performed prior to full CGA [38]. Due to its straightforwardness, as highlighted by Liuu, E. et al. [40], the G8 screening tool was the most reported screening tool that was used by the studies in our literature search. There was no justification as to why G8 was the most popular amongst our studies; however, G8 is recommended by the French National Cancer Institute due to its ease of implementation as well as its high sensitivity and specificity [37].

The latest advice from the SIOG is for more vigorous testing of G8, which produces better sensitivity compared to the VES-13 and Triage Risk Screening Tool (TRST) [39]. This is also supported by the recent study by Kenig, J. et al. [41], who compared the use of eight different frailty tools. Reviewing the studies, collectively we are not able to draw a definitive conclusion as to what the best tools are that should be included in a full CGA.

### 4.6. Comparisons to Parks RM et al., 2012

This systematic review is an update of the systematic review that was conducted by Parks, R.M et al. in 2012 [10]. Our findings have added evidence to suggest CGA can predict survival in breast cancer patients and assess quality of life and which treatment option would produce the best outcomes for patients. Parks, R.M et al., 2012, showed that there was not enough evidence to recommend CGA in early breast cancer patients [10]. We can conclude that the most important domain to be assessed is functional status due to its omnipresence across studies. From the evidence that has been discussed in this review, CGA should be offered to older breast cancer patients. The previous study was limited by the amount of evidence available at the time. This present systematic review was able to almost double the number of studies that were analysed and provides evidence as to the benefits that a full CGA should be conducted. Notably, we are still uncertain as to which tools to use within a full CGA.

### 4.7. Limitations

Some of the studies used the CGA in patients with a variety of tumour sites, and data specifically for breast cancer patients cannot be extracted. This has been highlighted throughout the text where this has occurred. Furthermore, most studies have been performed in Europe; there may be possible bias, meaning that results are not reflective of global practice.

## 5. Conclusions

This systematic review confirms that the CGA is useful in the setting of breast cancer in terms of predicting factors influencing survival, as an adjuvant to treatment decision-making and helping to maintain quality of life. Due to the heterogeneous methodology across studies, it remains unclear as to which tools should be included in a full CGA, and hence further work is required to answer this question.

## Appendix A

**Table A1:** General characteristics of included studies. (Acronyms: ADL = activities of daily living, iADL = instrumental activities of daily living, MMSE = Mini-Mental State Examination, GDS = Geriatric Depression Scale, MHI5 = Mental health index, KPS = Karnofsky performance scale, CCI = Charlson comorbidity index, VES-13 = Vulnerable ELDERS SURVEY 13, MNA = multi-nutritional assessment, MOB-T = mobility tiredness index, QLQ-C30 = EORTC core quality of life questionnaire, G8 = G8 screening tool, LOFS = Leuven oncogeriatric frailty score scale, ASA = American society of anaesthesiologists grade, CIRS-G = Cumulative illness rating score for geriatrics, QLQ-BR23 = quality of life questionnaire—breast cancer module, SOAP = Senior Adult Oncology Program geriatric assessment, ECOG = Eastern Cooperative Oncology Group, ESS = Exton–Smith Scale, MNA-SF = multi-nutritional assessment—short form, VAS = visual analogue scale, SPMSQ = Short Portable Mental Status Questionnaire, MOS-SF-36 = physical function index of medical outcomes—short study form).

Scheme 1	Country of Study	Date	Author	Study Type	Aims of Analysis	Level of Evidence	N	Age in Years	Cancer Type	Stage	Tools That Were Used
1	United Kingdom	Mar 2022	Munir, A. et al. [29]	Single-centre prospective study	Evaluating whether the use of self-administered CGA in older patients with breast cancer patients resulted in a change in their treatment.	3	101	≥65	Breast	Early stage	ADL, iADL, KPS, number of falls, BMOC, MHI-17, MOSS-SS, TUG.
2	France	May 2021	Boulahassass, R. et al. [18]	Single-centre retrospective study	Analysing the quality of life and CGA domains within 6 months in older adults receiving accelerating partial breast irradiation.	3	37	≥70	Breast	—	iADL, MMSE, QLQ-BR23, QLQ-C30 and global QOL.
3	Belgium	Sep 2020	Quinten, C. et al. [24]	Multicentre prospective study	Assessing the relationship between CGA and health-related quality of life in older patients with breast cancer.	3	109	≥75	Breast	Early stage	ADL, iADL, MMSE, MNA-SF, LOFS, and CCI.
4	France	Aug 2020	Liuu, E. et al. [15]	Single-centre prospective cohort study	Assessing the prognostic value of MPI for 1-year mortality in elderly cancer patients.	3	433	≥75	23% Prostate, 17% skin, 15% colorectal, 12% breast	—	ADL, iADL, MNA-SR, SPMSQ, ESS, CIRS.
5	Germany, Belgium, U.K.	Nov 2018	Speigl, L. et al. [19]	Multicentre prospective study	To compare the relationship between patient fitness/frailty status and survival to the local tumour environment in older patients with breast cancer.	3	58	≥70	Breast	—	ADL, iADL, MOB-T, MMSE, GDS-15, MNA-SF, VAS and CCI.
6	USA	Nov 2018	Owusu, C. et al. [28]	Cross-sectional study retrospective study.	Examining the racial differences in physical performance amongst older women who have recently been diagnosed with cancer.	3	135	≥65	Breast	Stage 1–3	LTPA, MMSE, GDS, MET-physical activity.
7	France	Apr 2018	Falandry, C. et al. [17]	Multicentre prospective study	Assessing CGA in older patients with breast cancer with multiple treatment options.	3	631	≥70	Breast	—	G8 and VES-13
8	U.K.	Sep 2017	Okonji, D. et al. [20]	Multicentre prospective study.	Evaluating a cohort of older women using CGA to determine whether fitness explained the apparent under-treatment.	3	326	≥70	Breast	Stage 1–3	ASA, II, ECOG, KPS, ADL, iADL, G8 and CCI
9	Germany, Belgium, USA	Feb 2017	Bailur, J.K. et al. [21]	Multicentre prospective study.	To investigate how immune cell biomarkers evolve in older patients with breast cancer.	3	56	≥70	Breast	—	G8 screening tool, LOFS, ADL, iADL, MOB-T, MMSE, GDS-15, MNA-SF and CCI.
10	USA	Nov 2015	Guerad, E.J. et al. [14]	Single-centre retrospective study	To evaluate oncology providers recognition of and response to falls in older patients with cancer.	3	528	≥65	62% Breast	—	Karnofsky performance status score, ADL, iADL.
11	U.K.	Apr 2015	Stotter, A. et al. [12]	Single-centre retrospective study	To estimate the 3-year survival rate in frail patients with early breast cancer and to inform treatment decisions	3	398	≥43	Breast	—	MMSE, ASA, GDS IV, iADL, BI and Charlson
12	Germany	Feb 2015	Denkinger, M.D. et al. [23]	Single-centre retrospective study.	Assessing the value of different assessments for predicting fatigue after radiotherapy in older breast cancer patients.	3	74	≥65	Breast	—	VES-13, KPS, EORTC-QLQ-C30 and cancer-specific CGA
13	U.K.	Jan 2015	Parks, R.M. et al. [25]	Single-centre polit study	Assessing CGA for early cancer breast patients ages 70 and over.	3	47	≥70	Breast	Stage 1, stage 2	EORTC, QLQ-C30, QLQ-BR23
14	France	Jan 2014	Blanc, M. et al. [16]	Single-centre retrospective study.	Evaluating the impact of GCA on the final therapeutic management of cancer in patients >70.	3	191	≥75	Breast 3.9%, lung 10.5%, colon 17.1%	—	MMSE, Mini GDS, MNA, ADL, iADL, Ki, CCI and CIRS-G
15	Italy	Feb 2013	Biganzoli, L. et al. [27]	Single-centre prospective study	Evaluating the role of cardiovascular health in predicting the presence of an abnormality with the CGA screening tool.	3	259	≥70	Breast 50%, colorectal 27%	—	ADL, CRIS-G, GDS, iADL, MMSE, and VES-13
16	USA	Nov 2013	Owusu, C. et al. [26]	Cross-sectional retrospective study	To assess racial differences in functional disability amongst older women with non-metastatic breast cancer	3	581	≥65	Breast	Stage 1–3	ADL, iADL, MMSE, GDS and CCI
17	USA	Apr 2012	Clough-Gorr, K.M. et al. [13]	Multicentre retrospective study	To investigate 5- and 10-year survival based on cancer CGA breast cancer patients amongst older women.	2	660	≥65	Breast	54% stage 1, 48% stage 2–3A	CCI, KPI, mini GDS, MMSE, MNA, ADL, CIRS-G, iADL, MOS-SF-36
18	France	Dec 2011	Freyer, G. et al. [22]	Multicentre retrospective observational study.	To describe the tolerance of women treated with adjuvant chemotherapy in patients aged >70 years.	3	110	≥70	Breast	—	ADL, MMSE, MNA, GDS

**Table A2:** Domains that were assessed and tools used in the studies (acronyms: ADL = activities of daily living, iADL = instrumental activities of daily living, MMSE = Mini-Mental State Examination, GDS = Geriatric Depression Scale, MHI5 = mental health index, KPS = Karnofsky performance scale, CCI = Charlson comorbidity index, VES-13 = Vulnerable Elders Survey 13, MNA = multi-nutritional assessment, MOB-T = mobility tiredness index, QLQ-C30 = EORTC core quality-of-life questionnaire, G8 = G8 screening tool, LOFS = Leuven ONCOGERIATRIC FRAILTY SCORE SCAle, ASA = American Society of Anesthesiologists grade, CIRS-G = cumulative illness rating score for geriatrics, QLQ-BR23 = quality-of-life questionnaire—breast cancer module, SOAP = Senior Adult Oncology Program geriatric assessment, ECOG = Eastern Cooperative Oncology Group, ESS = Exton–Smith Scale, MNA-SF = multi-nutritional assessment—short form, VAS = visual analogue scale, SPMSQ = short portable mental status questionnaire, MOS-SF-36 = physical function index of medical outcomes—short study form).

GA Domain	Tools Used (% of Studies Using)
Functionality	iADL (73.7); ADL (63.1); CCI (31.6); CIRS-G (10.5); ECOG (5.3)
Mobility/balance	MOB-T(5.3); LOFS (10.5)
Physical	MOS-SF-36 (5.3); ASA (10.5); ESS (5.3)
Socioeconomic	KPS (15.8)
Psychological	GDS-15 (36.6); MMSE (52.3); VAS (5.3); MHI5 (5.3); SPMSQ (5.3)
Nutritional status	MNA (15.8); MNA-SF (5.3)
Quality of life	QLQ-C30 (10.5); QLQ-BR23 (10.5)
Screening tools	VES-13 (15.8); G8 screening tool (10.5); SOAP (5.3)

**Table A3:** Statistical analysis of studies.

Study	Author	Findings	Statistical Analysis	Future Directions
1	Munir, et al. [29]	Self-administered frailty assessment may influence treatment recommendations. The results emphasise the importance and potential benefit of treatment choice in older patients with breast cancer.	Data were analysed using the Pearson’s chi-squared test. As the data were not normally distributed, differences were calculated using the Mann–Whitney U test. A multivariate logistic regression was performed to determine associations between CGA domains and change in treatment decision (*p* < 0.20).	Future research should focus on incorporating GA results to estimate more accurate the benefits of adjuvant chemotherapy in older patients with breast cancer.
2	Boulahassass, R. et al. [18]	The scores produced by CGA were decreased from the initial assessment compared with the CGA score performed 6 months later post-treatment, indicating a better quality of life for the patients following the treatment.	Statistical comparisons made using Student’s test or Mann–Whitney test for data that were continuous. All analyses were performed at a 5% alpha risk. Global QoL: baseline median 77.56 (SD 19.97); 1-month median 75.64 (SD 13.73), 3-month median 76.28 (SD 13.06); 6-month median 76.00 (SD 14.5).	Replicate the study on a larger patient cohort size to examine if the results could be replicated.
3	Quinten, C. et al. [24]	The study concludes that there are strong links between a CGA being undertaken and the patient’s quality of life post-treatment in regard to the treatment regimen they were on.	Correlation between EORTC QLQ-C30 functioning scales and GHS scale. GA measures were analysed at 3 time points using Spearman rank correlation coefficient. *p*-value was set at *p* < 0.05 calculated from Wald chi-squared test	CGA should be considered alongside the HRQOL regardless of treatment.
4	Liuu, E. et al. [15]	MPI based on CGA is shown to improve risk prediction of 1 year mortality and aid in cancer treatment intervention.	The time to event was plotted as Kaplan–Meier survival curve depending on the results of the MPI groups; compositions were then made using the log-rank test. Hazard ratio (HR) and 1-year mortality was determined by Cox proportional hazards. Univariate and multivariate models were used, with adjustments for age, sex and tumour site.	Studies focusing on whether MPI can help to predict survival rate of patients with breast cancer.
5	Speigl, L. et al. [19]	Fluorescence microscopy was used to investigate clinical outcomes compared to CGA evaluation. Those who the CGA determined to be fitter correlated with those who also have a higher abundance of CD3+ infiltrating cells, indicating better survival rates of the patients.	Correlations were assessed suing the non-parametric two-tailed (Spearman) correlation tests. Differences between groups were assessed with Mann–Whitney U tests, survival analysis was performed using the Kaplan–Meier method, and the log-rank test was applied. Significant relationships were considered at *p* < 0.05.	Further research includes; identifying patients using CGA that might not be appropriate for normal conventional treatment, but within a shorter time frame than the standard CGA timeframe.
6	Owusu, C. et al. [28]	CGA can be used as an initial indicator for cancer treatment to determine a poor physical performance prior to breast cancer treatment. CGA correctly indicated that those of African American (AA) ethnicity would have a poorer outcome compared to their white counterparts.	The chi-squared test or Fisher’s exact test was used to determine statistically significant results in the distribution between baselines of two groups. Univariate regression was used to examine the relation of race and other variables to determine statistical significance for poor physical status (*p* < 0.10).	Further research to investigate whether financial toxicity could also play a part in poorer outcomes with those from an AA heritage.
7	Falandry, C. et al. [17]	The study concludes that there has been an increase in the amount of CGA to aid with the treatment decision-making process. CGA can aid in determining which treatment regime might be optimal for patients over a certain age range.	Data were collected between October 4 and November 8 2011 by either face-face interviews or questionnaires. Qualitative data were presented as percentage and quantitative data were described as averages (mean, median, standard deviation, and range). Chi-squared test was used to compare baseline variables *p* < 0.05.	Further research is required to investigate the validity of CGA regimens that are being used.
8	Okonji, D. et al. [20]	CGA used to assess high risk of elderly women who would normally be in receipt of adjuvant chemotherapy, but were offered primary surgery, as the CGA predicted a better survival rate and outcome.	Two tailed *p*-values were calculated using Fisher’s exact test and *p* < 0.05 was considered significant. *p*-values as follows: breast surgery 0.0002, axillary surgery 0.0340, adjuvant radiotherapy 0.8195, chemotherapy 0.0001, HER2 positive (trastuzumab) 0.7451, ER-positive 0.7451.	Investigating the survival rate of women over 70 years old, who undergo primary surgery but do not receive chemotherapy.
9	Bailur, J.K. et al. [21]	CGA can be used as a fragility marker to measure the patient’s progression over the course of their treatment.	The Kruskal–Wallis test was used to compare biomarkers between more than two groups. The Mann–Whitney U test was used to compare data between two groups and Fisher’s exact test was used to determine the association between CMV and other variables. The study was a hypothesising study, and as such did not necessitate correction for multiple testing. All *p*-values were exploratory.	The immune biomarkers identified could be used in future research to better guide therapeutic management of patients with breast cancer.
10	Guerad, E.J. et al. [14]	There is a need to increase awareness of falls prevalence and consequences among oncology providers in order to provide timely interventions to reduce the risks associated with falls.	Percentages and frequencies were reported: 10% of patients had falls documented, 20% of patients had their gait assessed, 6% were referred for further assessment and 17% had vitamin D levels measured.	To increase oncologist awareness of the greater chance of falls for older patients with cancer. Implementing a falls assessment within the CGA.
11	Stotter, A. et al. [12]	CGA was shown to indicate good survival rates. Poor CGA was associated with a reduced survival score. CGA was recommended to complete before treatment commences and to aid with therapeutic choice.	The study’s characteristics were described using mean, median, range and percentages. The risk score was derived using logistic regression, by calculating probability of death within 3 years from the intercept and β-coefficients from all elements of the CGA. The Charlson index was used to develop the final risk score.	A larger prospective patient cohort on CGA should be conducted to help improve the assistance of treatment decision-making.
12	Denkinger, M.D. et al. [23]	CGA and frailty score were better indicators at predicting fatigue in a group compared to other variables in women with primary breast cancer.	Descriptive baseline statistics were analysed. Variables that were included in the models were chosen depending on their univariate correlation with both outcomes.	Further research to compare the current assessment to different outcomes, different time points, and different populations with alternative functional states.
13	Parks, R.M. et al. [25]	The study confirmed the feasibility of using CGA in a research setting.	Categorical data were described using percentages and frequency. Chi-squared test used to compare patient characteristics. Fisher’s exact test was used for smaller samples. T test was used for normal data distributed around the mean or Mann–Whitney test for data not normally distributed. All tests considered *p* < 0.05 to be significant.	More data will need to be gathered to definitively determine whether the components are required for a CGA.
14	Blanc, M. et al. [16]	CGA should be used alongside the oncologist to aid treatment regime.	Qualitative variables were described as percentages. Quantitative variables were described as means (SD), medians and ranges. Comparative analyses were determined with chi-squared test, Fisher’s exact test or Cochran–Armitage trend test. Relationship between mortality and comorbidity was assessed using univariate Cox regression analysis.	Conducting a prospective multicentre randomised studies to determine the impact of CGA to aid in potential treatment options.
15	Biganzoli, L. et al. [27]	Evaluating whether CHS can be used in the GCA. The use of CHS for CGA may be limited due to the stage of the disease of patients with breast cancer.	Patient characteristics were described using percentages and frequencies. 250 patients were recruited to provide a two-sided 95% confidence interval for accuracy of estimates with an equal width of 0.15 or closer. This was assuming the prevalence of impairment was equal to 60%.	Investigating whether CHS can replace VES-13 screening tool in a CGA.
16	Owusu, C. et al. [26]	Functional disability in older women with early breast cancer is highly prevalent in African American women compared to women of other races.	Bivariate analysis of all variables by race. The chi-squared test or Fisher’s exact test was used to determine statistically significant differences between the two groups. All *p*-values calculated were two-sided.	Investigating whether interventions to optimise functional status of at-risk groups (African American women) during and after cancer treatment is needed to help improve treatment tolerance and overall survival.
17	Clough-Gorr, K.M. et al. [13]	5- and 10-year survival rates can be indicated by a CGA being conducted. CGA should be used to aid in treatment regime regardless of age and stage of disease.	Descriptive statistics on all study variables were calculated. Bivariate distributions were evaluated between independent and mortality outcomes using Spearman correlations, chi-squared, log-rank test and Cochran–Armitage test. Five- and ten-year survival was analysed using Kaplan–Meier. Cox proportional hazards were used to predict five- and ten-year all-cause and breast-cancer-specific mortality.	Investigating the survival rate using C-SGA in various populations of older adults with different cancer types.
18	Freyer, G. et al. [22]	CGA is a useful tool that can help predict chemotherapy toxicities; however, performing CGA can be limited to geriatricians being available, specifically when treating older patients.	Data were descriptive in nature (mean and standard deviation for continuous data). Frequencies and percentages were calculated for categorical data. 95% confidence intervals were calculated when relevant.	Investigating the collaborative effect of performing CGA alongside the oncologist and geriatrician.
